# Taking the fear out of MRI safety queries: a modular educational intervention for the experts

**DOI:** 10.1007/s00261-025-05070-z

**Published:** 2025-06-16

**Authors:** Alexander Scott, Olivia Hallas, Blake Brandon, Mary Costello, Thomas Dang, Jake Maxfield, Swati Putcha, Jordyn Shah, Ayana Dambaeva, Rayan Abboud, Rekha Mody, Jenny Wu

**Affiliations:** Cleveland Clinic Foundation, Cleveland, USA

**Keywords:** Quality Improvement, Patient Safety, Inservice Training, Magnetic Resonance Imaging, Radiologist, Never Event

## Abstract

Expanding imaging indications and increasing patient complexity have created an increasing burden on radiologists for MRI safety related concerns and queries, especially related to implantable devices and foreign bodies. We present a single institution experience identifying deficiencies in radiologist MRI safety education and the subsequent implementation of a module-based training system. Using a pre- and post- intervention analysis, we demonstrated that 39% of institutional radiologists did not feel comfortable making MRI safety related decisions. Following a structure modular educational intervention, 95% of participants reported increased confidence in making MRI safety related decisions. We hope our institutional experience can highlight the need for MRI safety education and serve as a framework for future implementations preventing exam delays, inappropriate cancelations, or adverse safety events.

## Introduction

Magnetic resonance imaging (MRI) has been available as a clinical tool for the greater part of three decades and integrated into the education of thousands of currently practicing radiologists. However, expanding imaging indications and increasing complexity of patients have created an increasing burden of MRI safety related concerns and queries especially as it relates to implantable devices and foreign bodies. The early 2000s saw a boom in MRI utilization increasing to greater than 50% over a 15-year period [1]. At the same time, technological advances and safety research eliminated the historical contraindication to MRI in patient with implantable cardiac devices [2]. Newer conditional devices and older nonconditional devices can and do undergo MRI exams safely given proper precautions—a new burden of knowledge for the practicing radiologist.

Radiologists as the responsible MR imaging physician are tasked with a range of safety issues including approving or denying MRI exams on patients with implants, devices, metallic foreign bodies, answering questions related to MRI safety, and responding to contrast reactions. Having a solid foundation in MRI safety knowledge is critical to performing competently as a MR radiologist. Inadequate training and unfamiliarity with ACR MRI safety guidelines and protocols has the potential to create exam delays, inappropriate cancelations, or adverse safety events.

High-profile MRI safety events reaching the levels of sensational news headlines have brought increasing public scrutiny of MRI safety. As an example, the top read articles on Aunt Minnie for 2024 include stories regarding a SWAT raid into an MRI facility and an MRI non-compatible wheelchair entering the scanner causing patient injury [3]. These events generally fall under the category of “never events”—a broad list of medical preventable clearly identifiable medical events causing serious injury or death which should purportedly never occur [4]. Of the 29 “never events” defined by the National Quality Forum, “death or serious injury of a patient or staff associated with introduction of a metallic object into the MRI area” is the only radiology specific event [4]. These “never events” aside, there has been a general upward trend in MRI safety events reported to the FDA MAUDE database since 2018 [5, 6]. Furthermore, safety incidents are projected to be significantly under reported [7] highlighting the need proper prevention-based protocols.

A brief survey our single academic imaging institution demonstrated that 39% staff radiologists did not feel confident making decisions or performing tasks related to MRI safety. Additionally, 78% of the surveyed radiologists reported wanting more education, guidance and information on MRI safety.

While our single institution survey did not demarcate the underlying reasons for lake of confidence in MRI safety decisions, national surveys demonstrate a severe lack in MRI safety training amongst MRI-trained radiologists [8]. A brief literature review demonstrates a lack Radiologist-focused MRI safety educational frameworks to combat these national trends. We offer below our institutional educational implementation aimed at improving safety deficits in the hope it can serve as a foundation for novel future implementations.

## Methods

We utilized a 12-week Solutions for Value Enhancement (SolVE) curriculum focused on implementation of A3 analysis. The A3 was selected due to the abundance of institutional resources and expertise with the methodology. The A3 method, originally pioneered by Toyota in the 1960’s, focuses on efficient organization using a multi-step storyboarding method; topic areas include: Background, Current Condition, Goal, Root Cause Analysis, Countermeasures, Implementation/Plan and Sustain/Follow-up [9].

**Fig. 1:**
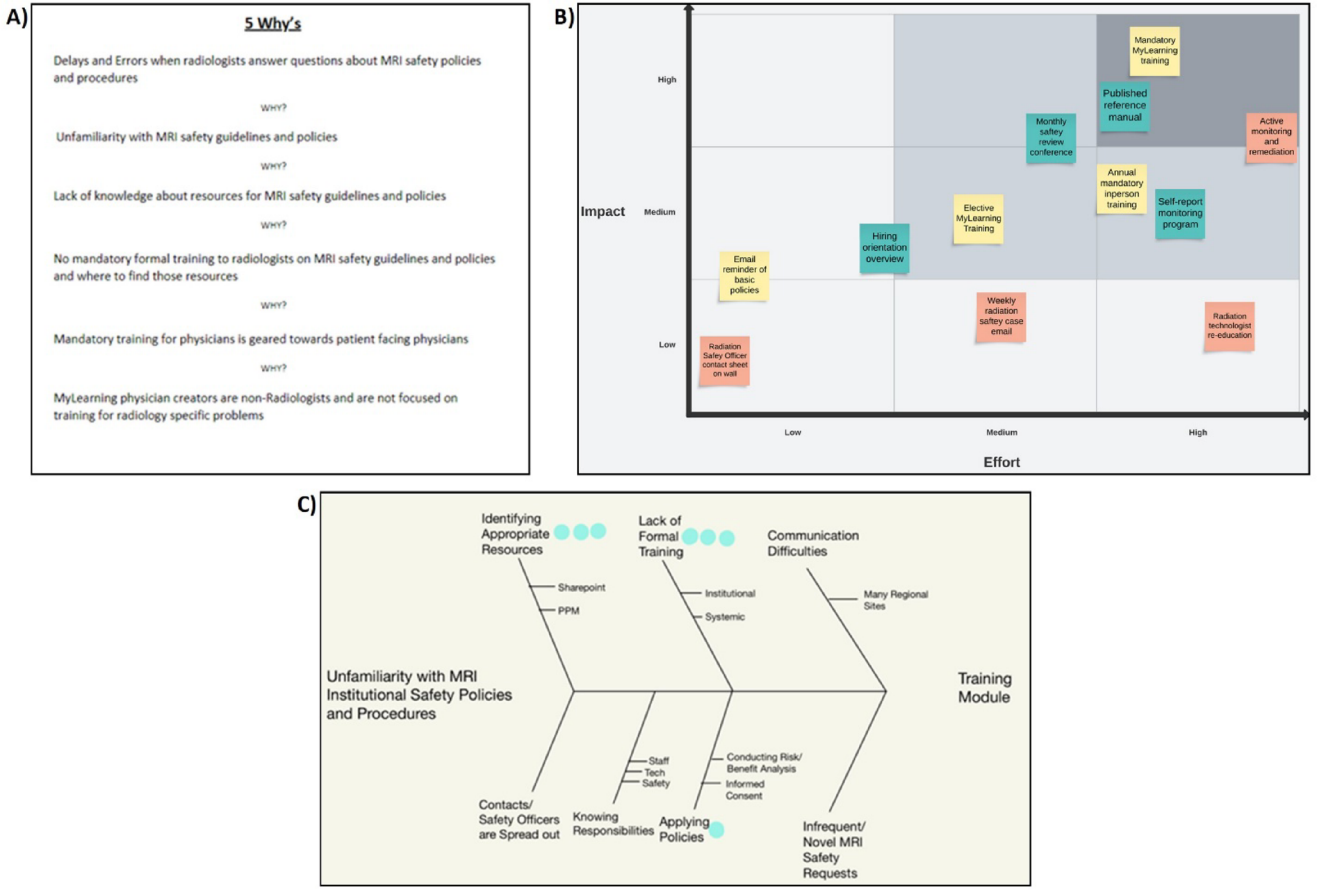
Materials demonstrating components of the A3 process. **A** A 5 Why’s methodology chart composed as part of our root cause analysis to determine underlying cause of lack of radiologist confidence in MRI safety decision making. **B** An impact effort matrix derived to help guide the optimal educational intervention. **C** A Fishbone/Ishikawa diagram used as part of the root cause analysis

**Fig. 2:**
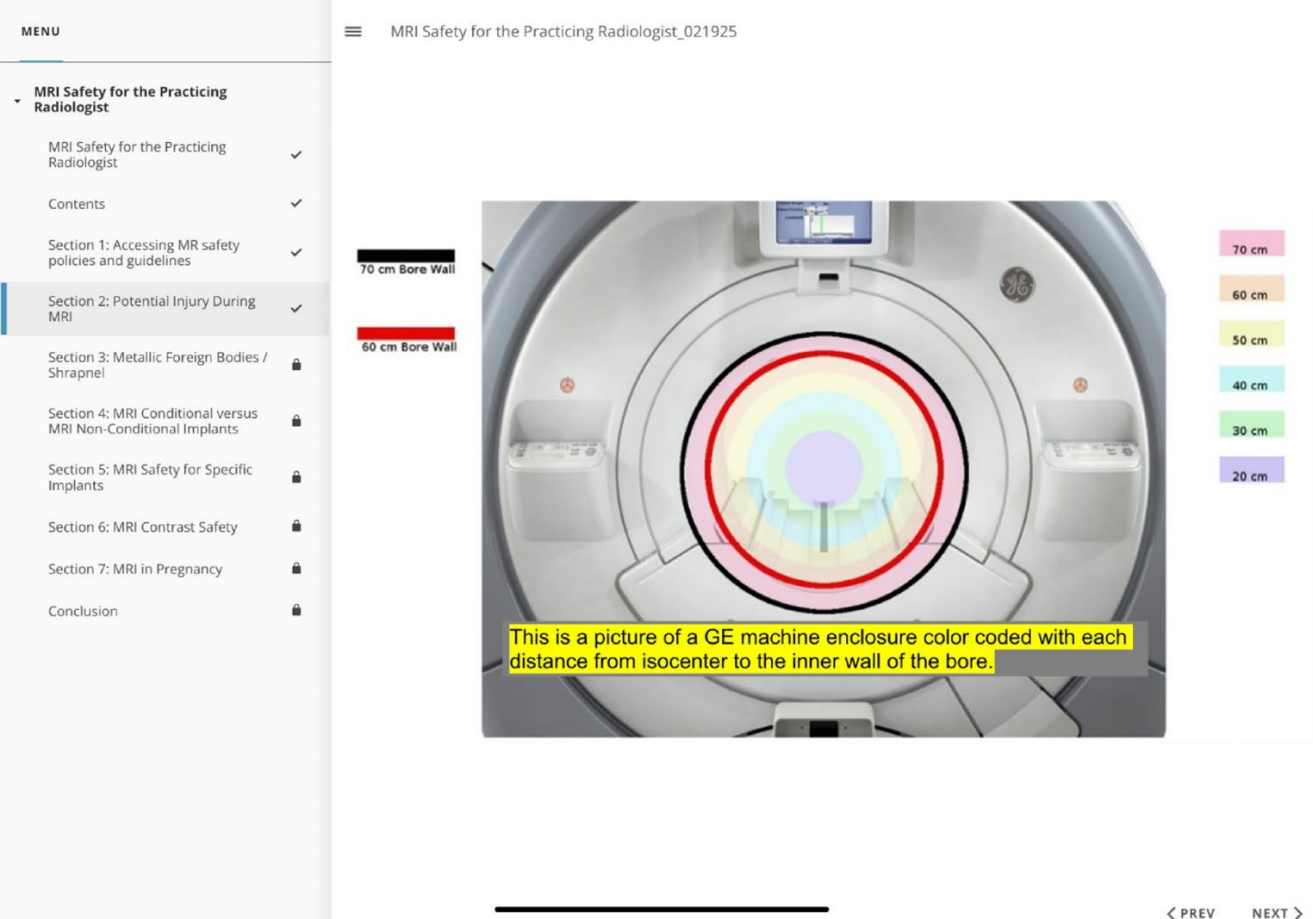
Example figure from the implemented safety module demonstrating isocenter distances within a GE MRI scanner as they relate to static magnetic field strength

To delineate our *current conditions* a survey was conducted to assess institutional knowledge and confidence in MRI safety topics. Survey participation was compulsory as part of mandatory yearly compliance trainings and all institution employed in-training and staff radiologists participated. Based on these survey results our *goal* was to increase radiologist subjective and objective confidence in MRI safety related tasks. Process maps of commonly reported MRI safety deficits were created with points of interest for possible areas of system failure identified. A *root cause analysis* was conducted using the 5 Why’s methodology and a Fishbone diagram (Fig. 1A and C). Based on an effort-impact matrix, a mandatory, recurring MRI Safety training module was recommended as the highest impact *countermeasure* for deficiencies in radiologist knowledge (Fig. 1B). A training module for all institution radiologists was subsequently developed and *implemented* with the assistance of our institution’s MRI safety team as part of yearly compulsory compliance trainings. An example image from the module is provided in Fig. 2. Recertification and yearly completion of the module provides *follow-up* to ensure knowledge retention  .

**Fig. 3:**
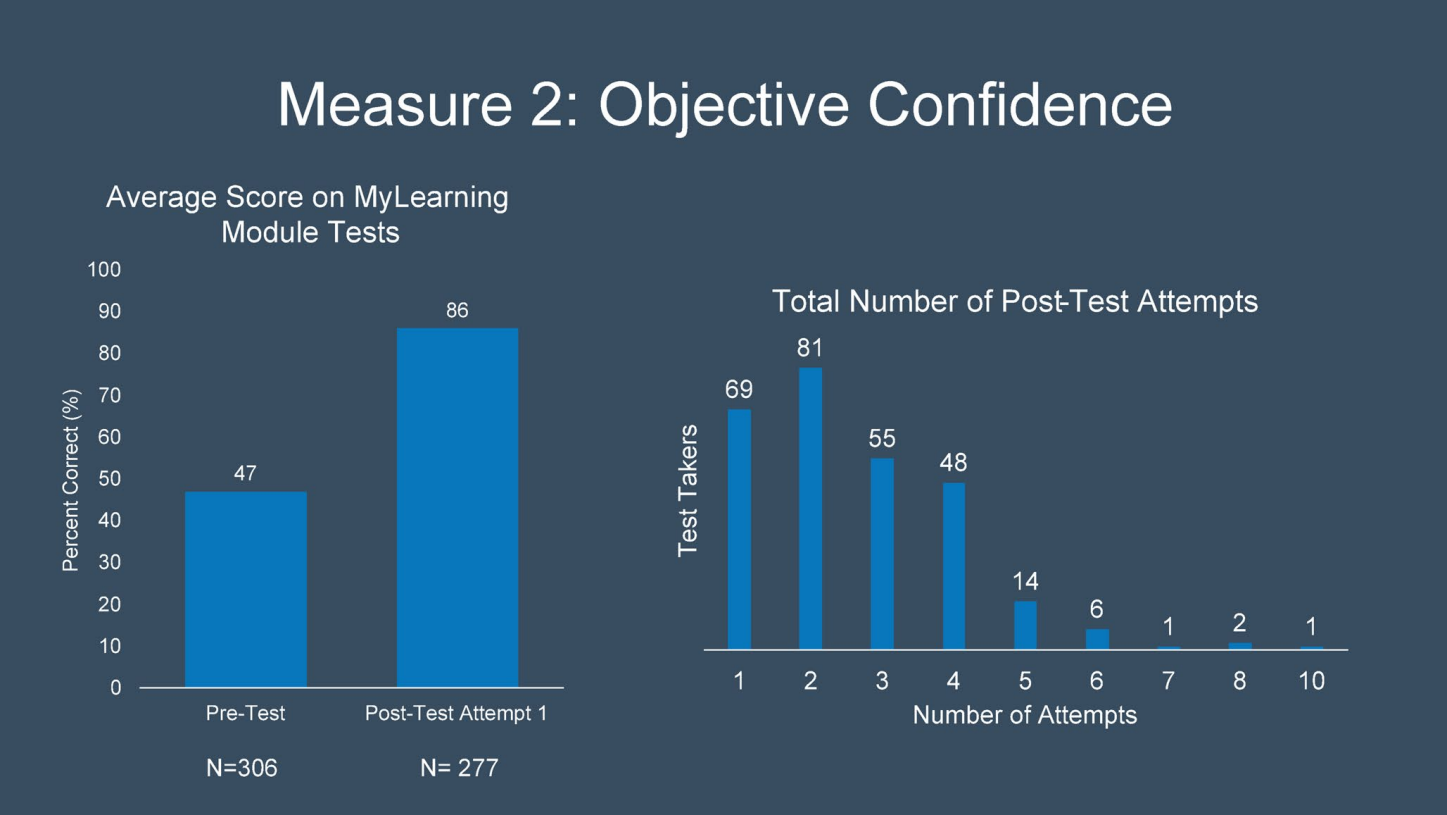
Pre and post-test objective confidence measures demonstrating increased proficiency following module implementation

The training module was divided into seven sections: (1) Accessing MR Safety Policies and Guidelines, (2) Potential Injury During MRI, (3) Metallic Foreign Bodies/Shrapnel, (4) MRI Conditional Versus MRI Non-Conditional Implants, (5) MRI Safety for Specific Implants, (6) MRI Contrast Safety, and (7) MRI in Pregnancy.

To assess the module implementation and impact on radiologist confidence in completing MRI safety related tasks, outcomes were determined by two measures:

Measure 1: Self-reported confidence (“yes” response to increased confidence on post-training survey).

Measure 2: Pre- and post-test knowledge scores, and number of attempts needed to achieve a perfect score.

Pre and post-test questions examined topics such as application of static and spatial gradient magnetic fields, solutions to problems such as peripheral nerve stimulation, and non-conditional cardiac implant policies. Module participation was made compulsory as part of yearly institutional certification/trainings to ensure response of all radiologists across institutional practice environments and training levels.

## Results

From September through December 2023, 292 in-training and staff radiologists completed the training module and subsequent post-intervention assessment. Of those who completed the module and tests, 95% reported that the module increased their confidence level in making decisions or performing tasks related to MRI safety. Pre intervention test scores (M = 47, SD = 16.17) and post intervention first test scores (M = 85.02, SD = 13.30) demonstrate significant improvement in post intervention MRI safety knowledge t(290) = 35.9, *p* < 0.001. The median attempts for a perfect post-intervention score were 2 (IQR 2)(Fig. 3).

## Discussion

The mandatory MRI safety training was well received and led to measurable improvements in both subjective confidence and objective performance on safety-related tasks. These results support the effectiveness of a structured, formal educational intervention in addressing identified knowledge gaps in radiologist MRI safety preparedness. Longitudinal follow-up with yearly training and assessment, will hopefully only further interventional efficacy.

Importantly, the A3 methodology provided a clear and efficient framework for identifying root causes, selecting high-impact interventions, and implementing change in a complex healthcare environment. By combining root cause analysis, stakeholder input, and decision tools such as the effort-impact matrix, we were able to prioritize a sustainable solution that balanced educational burden with clinical benefit. This structured approach may serve as a model for other radiology departments or academic institutions facing similar challenges in safety education or professional development.

MRI safety is a highly nuanced and evolving area, particularly as device technology advances and patient complexity increases. Despite its critical nature, MRI safety education often remains inconsistent or informal across training programs. Our internal survey data—where 39% of radiologists reported a lack of confidence in MRI safety decision-making—highlights the extent of this educational gap even among experienced clinicians. Moreover, the 78% of respondents seeking further guidance reflects a strong demand for structured, continuing education in this area.

While institutional results are promising, this was single institution implementation, which may limit the generalizability of the results. Institutional culture, resources, and baseline safety practices can vary significantly across settings, potentially influencing both the implementation process and outcomes. As such, multicenter validation is recommended to establish the efficacy of structured MRI safety education across diverse practice environments.

Given the universal role radiologists play in MRI safety oversight—and the increasing complexity of implantable devices—it is reasonable to suspect that similar knowledge gaps exist at many other institutions. As such, national or specialty-wide efforts to standardize MRI safety training, including integration into maintenance of certification (MOC) programs or ACR-endorsed CME, could further elevate safety standards across the field.

In conclusion, targeted, structured MRI safety education using quality improvement methodologies such as A3 analysis can significantly enhance radiologist preparedness and institutional safety culture. As MRI utilization continues to rise, ensuring that radiologists possess up-to-date, evidence-based safety knowledge is not only essential but also actionable through thoughtful and scalable interventions.

## Data Availability

No datasets were generated or analysed during the current study.
